# Engineering Vascularized Islet Macroencapsulation Devices: An *in vitro* Platform to Study Oxygen Transport in Perfused Immobilized Pancreatic Beta Cell Cultures

**DOI:** 10.3389/fbioe.2022.884071

**Published:** 2022-04-19

**Authors:** Fernandez S. A., Champion K. S., Danielczak L., Gasparrini M., Paraskevas S., Leask R. L., Hoesli C. A.

**Affiliations:** ^1^ Department of Chemical Engineering, McGill University, Montréal, QC, Canada; ^2^ Human Islet Transplant Laboratory, McGill University Health Centre, Montréal, QC, Canada; ^3^ Department of Surgery, McGill University Health Centre, Montréal, QC, Canada; ^4^ Department of Biomedical Engineering, McGill University, Montréal, QC, Canada

**Keywords:** type 1 diabetes, immobilized culture, artificial vascularization, oxygen mass transport, oxygen model, beta cells, encapsulation

## Abstract

Islet encapsulation devices serve to deliver pancreatic beta cells to type 1 diabetic patients without the need for chronic immunosuppression. However, clinical translation is hampered by mass transport limitations causing graft hypoxia. This is exacerbated in devices relying only on passive diffusion for oxygenation. Here, we describe the application of a cylindrical *in vitro* perfusion system to study oxygen effects on islet-like clusters immobilized in alginate hydrogel. Mouse insulinoma 6 islet-like clusters were generated using microwell plates and characterized with respect to size distribution, viability, and oxygen consumption rate to determine an appropriate seeding density for perfusion studies. Immobilized clusters were perfused through a central channel at different oxygen tensions. Analysis of histological staining indicated the distribution of viable clusters was severely limited to near the perfusion channel at low oxygen tensions, while the distribution was broadest at normoxia. The results agreed with a 3D computational model designed to simulate the oxygen distribution within the perfusion device. Further simulations were generated to predict device performance with human islets under *in vitro* and *in vivo* conditions. The combination of experimental and computational findings suggest that a multichannel perfusion strategy could support *in vivo* viability and function of a therapeutic islet dose.

## Introduction

A variety of pancreatic islet macroencapsulation systems are currently being explored to deliver therapeutic cells to type 1 diabetic patients. Diverse approaches and encapsulation biomaterials have been developed with the aim of increasing graft longevity and function. Ideally, an effective macroencapsulation system would eliminate the patient’s need for immunosuppressive drugs and exogenous insulin to achieve long term blood glucose normalization. However, a major obstacle plaguing these strategies is inadequate oxygenation throughout the cell-laden system. Oxygen diffusion gradients within thick constructs lead to regions of hypoxia, causing cell necrosis and functional impairment (Dionne et al., 1993; Olsson and Carlsson, 2006; Liljebäck et al., 2016).

As predicted by analytical modelling, high surface-area-to-volume ratios are required to achieve adequate oxygenation across encapsulated cells via passive diffusion (Fernandez et al., 2014; Papas et al., 2016). However, this would necessitate prohibitively large macrocapsules not feasible for surgical implantation. For example, Papas et al. calculate a required surface area on the order of 1,000 cm^2^ to adequately oxygenate an islet sheet device at physiological oxygen tensions (Papas et al., 2016). Given these predictions, it is evident that passive oxygen diffusion alone is insufficient to advance the efficacy of islet macroencapsulation designs.

There are several notable solutions currently proposed to directly oxygenate subcutaneous macroencapsulation devices. A prominent example is the βAir device developed by Beta-O2 Technologies, which features a dedicated oxygen chamber supplying oxygen gas to a cell-laden chamber (Neufeld et al., 2013). While delivering promising results in small animal tests, device performance remains to be demonstrated at the human scale. A limitation of this design is the need for regular maintenance to refuel the oxygen gas. A more recent solution was proposed by Wang et al., who have designed an inverse-breathing encapsulation device (iBED) that utilizes lithium peroxide particles as oxygen carriers (Wang et al., 2021). Similar to the βAir device, promising results have been obtained in small animal models. However, periodic device maintenance may be required to refill the oxygen carriers. Additionally, Komatsu et al. proposed an oxygen transporter device that utilizes ambient air to increase the oxygen tension in a subcutaneous transplant site (Komatsu et al., 2018). This approach circumvents the need for oxygen refueling and has shown to improve encapsulated cell survival in small animal studies. A disadvantage shared with the βAir approach is the need for external access ports for oxygen transport, which introduces potential routes for infection and patient discomfort. Generally, subcutaneous devices face scale-up challenges due to size limitations.

Alternatively, vascular prosthesis strategies offer the potential for enhanced oxygenation via blood convection, as well as long-term graft functionality without the need for device maintenance. Considering this, we have previously highlighted the need for robust *in vitro* platforms to study blood-device interactions and predict *in vivo* performance (Fernandez et al., 2021). We fabricated a system that comprises a perfusable culture device housing a cell-laden hydrogel (Fernandez et al., 2021). This hydrogel is irrigated with artificial vasculature to permit fluid convection. Using this platform, we have shown that alginate-encapsulated islet-like clusters (ILCs) exhibited higher cell survival under perfusion when contrasted with their static counterparts (Fernandez et al., 2021). This platform can be further used to assess factors impacting cell survival and function to better predict *in vivo* behaviour. The objective of the current work was to model and investigate the oxygen distribution and hypoxic effects in a perfused single-channel device. This was achieved by characterizing the oxygen consumption rate (OCR) of the ILCs and assessing their performance under different oxygen tensions. Computational modelling was also performed to predict the oxygen distribution within the device and evaluate the effect of different design parameters to guide the development of vascularized islet encapsulation systems.

## Materials and Methods

### Adherent Cell Culture

Mouse insulinoma 6 (MIN6) pancreatic beta cells were kindly provided by Dr. James D. Johnson, University of British Columbia (originally acquired from Dr. Jun-ichi Miyazaki, Osaka University). MIN6 cells in complete medium were seeded in tissue culture treated polystyrene flasks at a density of 4–8 × 10^5^ cells/cm^2^. Complete medium consisted of Dulbecco’s Modified Eagle medium (DMEM; Gibco #10313-021, Thermo Fisher Scientific) supplemented with 10% fetal bovine serum (FBS; HyClone #SH3039602, Fisher Scientific), 1% penicillin/streptomycin (Gibco #15140-122, Thermo Fisher Scientific), 1% L-glutamine (Gibco #25030-081, Thermo Fisher Scientific), and 0.1% 2-mercaptoethanol (Fisher Chemical #O3446I, Fisher Scientific). Cells were cultured in an aseptic incubator at 37°C and 5% CO_2_. Medium changes were performed every 48 h. Cells were passaged at 90% confluency using 1X TrypLE Express Enzyme (Gibco #12605-028, Thermo Fisher Scientific).

### Generation of Islet-like Cell Clusters

Adherent MIN6 cells were passaged twice before seeding in AggreWell^™^400 24-well plates (#34415, STEMCELL Technologies) to generate islet-like clusters (ILCs). The following seeding densities were used: 2.4 × 10^5^ cells/microwell (200 cells/ILC), 6 × 10^5^ cells/microwell (500 cells/ILC), 1.2 × 10^6^ cells/microwell (1,000 cells/ILC), 2.4 × 10^6^ cells/microwell (2,000 cells/ILC), 3.6 × 10^6^ cells/microwell (3,000 cells/ILC). Cells were cultured for 48 h in complete medium at 37°C and 5% CO_2_. Cell seeding and ILC harvesting steps were performed according to the AggreWell^™^400 manufacturer’s protocol.

### Live/Dead Staining

ILCs harvested from each macrowell of an AggreWell^™^400 plate were immediately transferred to a new well in a 48-well plate (∼1,200 ILCs/well). A live/dead staining solution was prepared with final concentrations of 5 µM calcein-AM (Invitrogen #C1430, Thermo Fisher Scientific) and 5 µM ethidium homodimer-1 (#ab145323, Abcam) in phosphate buffered saline (PBS; Gibco #21300025, Thermo Fisher Scientific). To each well, 150 µL of staining solution were added. Samples were incubated in the dark at room temperature for 30 min and then imaged using an Olympus IX81 inverted system microscope and MetaMorph^®^ acquisition and analysis software.

### Oxygen Consumption Rate Measurements

ILCs from each of the five seeding density conditions described above were harvested from one macrowell of an AggreWell^™^400 plate and immediately transferred to a 15-mL centrifuge tube and allowed to settle (∼3,000 ILCs/tube). Once settled, the ILCs were resuspended in 600 µL of DMEM and divided evenly into 1.5 mL tubes, each containing 200 µL (∼1,000 ILCs/tube). The OCR was determined as per previously reported methods (Papas et al., 2007a; Papas et al., 2007b). Briefly, the OCR was measured on a calibrated Instech Fluorescence Lifetime Micro Oxygen Monitoring System (Instech Laboratories, Plymouth Meeting, PA; FOL/C2T175PFOL) using Amplifier NeoFox software. Immediately before measuring, the DMEM was removed from the ILCs and replaced with 200 µL of serum-free DMEM. The sample was mixed to ensure a homogeneous solution and 170 µL was transferred to each reading chamber where the oxygen partial pressure (pO_2_) was monitored. Once the measurement was complete, the ILCs were carefully collected and stored at −80°C in preparation for DNA quantification.

### DNA Quantification

ILCs were lysed by performing a freeze-thaw cycle and sonicating for 15 s on ice. DNA quantification was performed using the Quant-iT™ PicoGreen™ dsDNA Assay Kit (Life Technologies, Carlsbad, CA; Cat #P7589) according to the manufacturer’s protocol.

### Oxygen Consumption Rate Calculation

The OCR calculation was performed as previously reported by Papas et al. by determining a linear region of the measured pO_2_ versus time plot and calculating the slope (Papas et al., 2007b). This was then normalized to the DNA content to give an OCR/DNA ratio.

### Encapsulation of Islet-like Clusters

A stock solution of 2.5% alginate (Manugel GHB, IFF Nutrition & Biosciences (formerly FMC Biopolymer); 60–65% guluronic acid; 1% solution viscosity 50–100 mPa s) and a stock suspension of 0.5 M calcium carbonate (CaCO_3_; Avantor #1301-01, VWR) were both prepared in pH 7.4 HEPES-buffered saline (Fisher BioReagents #BP310 and #BP3581, Fisher Scientific) and autoclaved at 121°C for 30 min. Glucono-δ-lactone (GDL; Sigma-Aldrich #G4750, MilliporeSigma) was dissolved in HEPES-buffered saline and sterilized with a 0.2-µm nylon syringe filter (Fisherbrand™ #09-719C, Fisher Scientific) immediately before use. Using the above reagents, an alginate hydrogel mixture was prepared with final concentrations of 2% alginate, 30 mM CaCO_3_, and 60 mM GDL. A 9:1 volume ratio of alginate-to-medium was maintained.

MIN6 ILCs were generated using AggreWell^™^400 plates as previously described and added to the gel mixture. A seeding density of 1.2 × 10^6^ cells/macrowell was used to achieve 1,000 beta cells/ILC. GDL was mixed in immediately prior to casting to trigger internal gelation with a final concentration of 1,000 ILCs/mL gel. The gel mixture was loaded via syringe into a cylindrical perfusion device outfitted with a 2-mm diameter stainless steel rod as a vascular template. The device was incubated at room temperature for 60 min for adequate gelation. The vascular template was then removed, leaving a central hollow channel, and the device was connected to a tubing loop for perfusion culture. Device assembly is described in detail in our previous work (Fernandez et al., 2021). A general schematic of the device geometry is provided in Figure 3A.

### Perfusion Culture of Encapsulated Cells

Perfusion of culture medium was established using a peristaltic pump set up inside an incubator at 37°C. O_2_ levels were set to 2% (hypoxia), 10% (arterial), or left at ambient 18.6% (*in vitro* normoxia); CO_2_ was maintained at 5% and N_2_ made up the balance (Wenger et al., 2015). Hypoxic and arterial conditions were established using a BioSpherix ProOx C21 system to control CO_2_ and O_2_. *In vitro* normoxic conditions were established using a regular cell culture incubator with CO_2_ control. Culture medium was pre-incubated at the desired O_2_ tension for 24 h before beginning perfusion. A culture medium flow rate of 3 mL/min was selected to mimic laminar blood flow through a medium to small artery 2 mm in diameter (Reynolds number *Re* < 50). The medium was drawn from a 30-ml reservoir of DMEM and perfusion was run for 48 h.

### Histological Staining and Microscopy

Fixed samples were sent to a core facility of McGill University for paraffin embedding, sectioning, deparaffinization and staining. Sections of 6-µm thickness were taken orthogonal to the perfusion channel. Per device, 8 stained sections were acquired: 2 × hematoxylin and eosin (H&E) for general tissue visualization, 2 × anti-cleaved caspase-3 (CC3; New England BioLabs, 9,661) to visualize apoptotic cells, 2 × anti-hypoxia-inducible factor 1-alpha (HIF-1α; Novus Biologicals, NB100-479) to visualize oxidative stress, and 2 × anti-Ki67 (clone B56) (#3168022D, Fluidigm Corporation) to visualize proliferative cells. Ki67-stained samples were omitted from analysis due to patchy staining results. Imaging was performed using a Zeiss Axio 200M automated inverted microscope and Zen Pro acquisition and analysis software. Images were taken at 20X magnification and stitched to assemble a complete cross-section of the sample.

### Computational Modelling

COMSOL Multiphysics 5.5 and MATLAB™ 2021b were used to develop and render models simulating the perfusion device. The model uses two sets of physics options: 1) laminar flow and 2) transport of dilute species (oxygen). The boundary conditions prescribed for 1) are: laminar flow at the inlet with a known flow rate; zero pressure at outlet (results in gauge pressure profile). For (2), the boundary conditions are: a fixed inlet concentration of oxygen; advection dominant transport at the outlet (pre-programmed in COMSOL). All models were solved in COMSOL and post-processed in MATLAB.

The perfusion device geometry was replicated in the COMSOL interface and assigned to different materials (cell culture medium or cell-laden alginate). The physical properties of the cell culture medium were assumed to be the same as water at 37°C. The cell-laden alginate was modelled as a porous medium domain with modified Michaelis-Menten oxygen consumption kinetics supported and developed in current literature as shown in Table 1 (Buchwald, 2011).

**TABLE 1:** Computational model parameters for a perfused macroencapsulation device.

Parameter	Value/Equation	Source	Description
$R_{max,O_{2}}$ (human islets)	0.034 mol s^−1^ m^−3^	Avgoustiniatos et al. (2007), Buchwald (2009); (2011), Coronel et al. (2017)	Maximum oxygen consumption rate of human islets
$R_{max,O_{2}}$ (MIN6)	0.129 mol s^−1^ m^−3^	Experimental	Approximate maximum oxygen consumption rate of MIN6 cells (based on measured 780 nmol/min/mg)
$c_{cr}$	0.1 mmHg	Johnson et al. (2009), Suszynski et al. (2014), Cao et al. (2020)	Partial pressure at which cell necrosis is observed
$K_{MM,O_{2}}$	0.62 µM	Johnson et al. (2009), Buchwald (2011), Avgoustiniatos and Colton (1997)	Michaelis-Menten coefficient for oxygen diffusion (0.44 mmHg)
$K_{alginate}$	∼10^−15^ cm^2^	Refojo (1965), Albro et al. (2007), Xin et al. (2016)	Permeability of alginate, assumed in range of common hydrogels
*ε*_*alginate*_	0.98	Calculated	Porosity, based on 2% alginate
*X*_*MIN6,experimental*_	0.010	Calculated	Cell fraction (v/v), based on 1,000 MIN6 ILCs/mL gel with a mean ILC diameter of 269 µm
*X*_*islets,human-scale*_	0.058	Calculated (Buder et al., 2013)	Cell fraction (v/v), based on 600,000 islet equivalents (IEQ; mean islet diameter 150 µm) in an 18-mL device
*V*_*tot*_	18 mL	Experimental	Total gel volume in the device
*D*_*water*_	3.1 × 10^−9^ m^2^ s^−1^	Buchwald (2009)	Diffusivity of oxygen in water (or cell culture medium)
*D*_*alg*_	2.54 × 10^−5^ cm^2^ s^−1^	Mehmetoglu et al. (1996), Cristea et al. (2020)	Diffusivity of alginate
*D*_*tissue*_	1.24 × 10^−5^ cm^2^ s^−1^	Johnson et al. (2009), Buchwald (2011)	Diffusivity of tissue (islets)
*D*_*eff*_	$D_{a\mathrm{lg}}\left(1-X\right)+D_{tissue}X$	Calculated	Effective diffusivity of cell-laden matrix based on weighted ratio of alginate and tissue
*µ*_*alginate*_	0.4 Pa s	Kong and Mooney (2011)	Viscosity of fully cross-linked alginate
*Q*	3 mL min^−1^	Experimental	Flow rate in system (subject to change)
*c*_*in*_	0.198 mol m^−3^	Experimental	Inlet oxygen concentration, experimental shown (140 mmHg O_2_; subject to change)
*H*_*O2*_	$∼1.4x10^{-3}\frac{mol}{m^{3}mmHg}\;$ ^a^	Linstrom and Mallard (1997)	Henry’s constant for converting mol/m^3^ to mmHg at 37°C for water (or cell culture medium)

a This value is comparable to that found in literature for islet oxygenation models at 35°C (Buchwald, 2011).

The system was solved as one fully coupled model in COMSOL using a convection-diffusion model (Eq. 1) with a reaction term (Eq. 2) representing oxygen consumption. This approach is consistent with analytical models without advection (Dulong and Legallais, 2007) or using oxygen partial pressure instead of oxygen concentration in the convection-diffusion equation (Komatsu et al., 2017).

$$-D_{eff}\nabla^{2}c_{O_{2}}+u\cdot \nabla c_{O_{2}}=R$$ (1)

$$R=\left[-R_{max,O_{2}}\;\frac{c_{O_{2}}}{c_{O_{2}}+K_{MM,O_{2}}\;}\;\delta \left(c_{O_{2}}>c_{cr}\right)\right]X$$ (2)

Where *D*_*eff*_: effective diffusivity of cell-laden alginate; *c*_*O2*_: concentration of oxygen; *c*_*cr*_: critical concentration of oxygen below which cell necrosis occurs; *u*: velocity field; *R*: oxygen consumption; *R*_max*,O2*_: maximum oxygen consumption rate; *K*_*MM,O2*_: Michaelis-Menten coefficient for oxygen diffusion; *δ*: Heaviside function; and *X*: cell fraction.

All single channel models were solved in a 2D axisymmetric system while 2-channel systems were solved in 3D. The main controlled parameters of the simulation are the flow rate *Q*, cell fraction *X*, and channel geometry. The model assumes rigid device walls, Newtonian fluid, fully developed laminar flow, no thermal diffusion, no pressure loss, no backflow, negligible body forces, and a homogeneous cell fraction throughout the hydrogel. A “normal” mesh size was used for an optimal trade-off between computation time and visual quality. A relative tolerance of 1 × 10^−6^ (0.0001% error) was selected as the convergence criterion for all oxygen maps. A relative tolerance of 1 × 10^−5^ (0.001% error) was selected for the parametric analyses to reduce computation time. The solving method employed was a tolerance-based direct solver. The Heaviside function (*δ*) serves to set the oxygen consumption rate to zero in regions below the necrotic oxygen partial pressure (*c*_*cr*_; see Table 1), it also helps in avoiding negative concentrations and divergence (Komatsu et al., 2017).

All assumed model parameters are listed in Table 1. The oxygen consumption rate *R*_max_ was either found experimentally for MIN6 or taken from literature for human islets. The cell fraction *X* was calculated using a volume fraction *V*_*cells*_ */V*_*total*_ assuming islets are spheres with radius *r*. The effective diffusivity of cell-laden alginate *D*_*eff*_ was calculated as a weighted average of the diffusivity in cell tissue *D*_*tissue*_ and in alginate *D*_*alg*_ (Eq. 3). The Henry’s constant used is for water at 37°C.

$$D_{eff}=D_{tissue}X+D_{alg}\left(1-X\right)$$ (3)

An analysis of the model parameters listed in Table 1 was performed using a parametric sweep in COMSOL Multiphysics. Default parameter values were assigned to reflect the experimental conditions used during the perfusion studies described above. Individual parameter values were varied over a selected range and plotted at six points—three within 1 mm of the device entry and three within 1 mm of the exit. These points were located at different radial positions between the perfusion channel and the gel periphery (0, 250 μm, 500 µm). A “coarser” mesh size was applied near the gel periphery and a “normal-fine” mesh was applied closer to the perfusion channel to resolve the kinetics. These points also facilitate an assessment of the effects of channel length on theoretical oxygenation.

### Image Analysis and Statistics

MIN6 ILC sizes were measured using the manual freeform selection tool in ImageJ software. Size distributions, volume mean moment, and standard deviations were calculated in Microsoft Excel and plotted in GraphPad Prism 5.01.

MIN6 viability was analyzed by measuring fluorescence intensity with Fiji software. Mean values and standard deviations were calculated in Microsoft Excel and plotted in GraphPad Prism. One-way analysis of variance (ANOVA) and the Tukey multiple comparisons test were performed in GraphPad Prism to compare the means of the sample conditions (*α* = 0.05).

Oxygen consumption rates normalized to DNA content were calculated in Microsoft Excel. One-way ANOVA was performed in GraphPad Prism to compare the means of the sample conditions (*α* = 0.05).

Due to large file sizes, stitched histology images were resized to 50% and exported from Zen Pro software. ILC size, distance, and mean grey value measurements were acquired using Fiji software and analyzed in Microsoft Excel and GraphPad Prism. Statistical analysis of mean grey values was performed using a one-way ANOVA followed by the Tukey multiple comparisons test (*α* = 0.05).

## Results

### Size Distribution and Survival of MIN6 Islet-like Clusters

**FIGURE 1:**
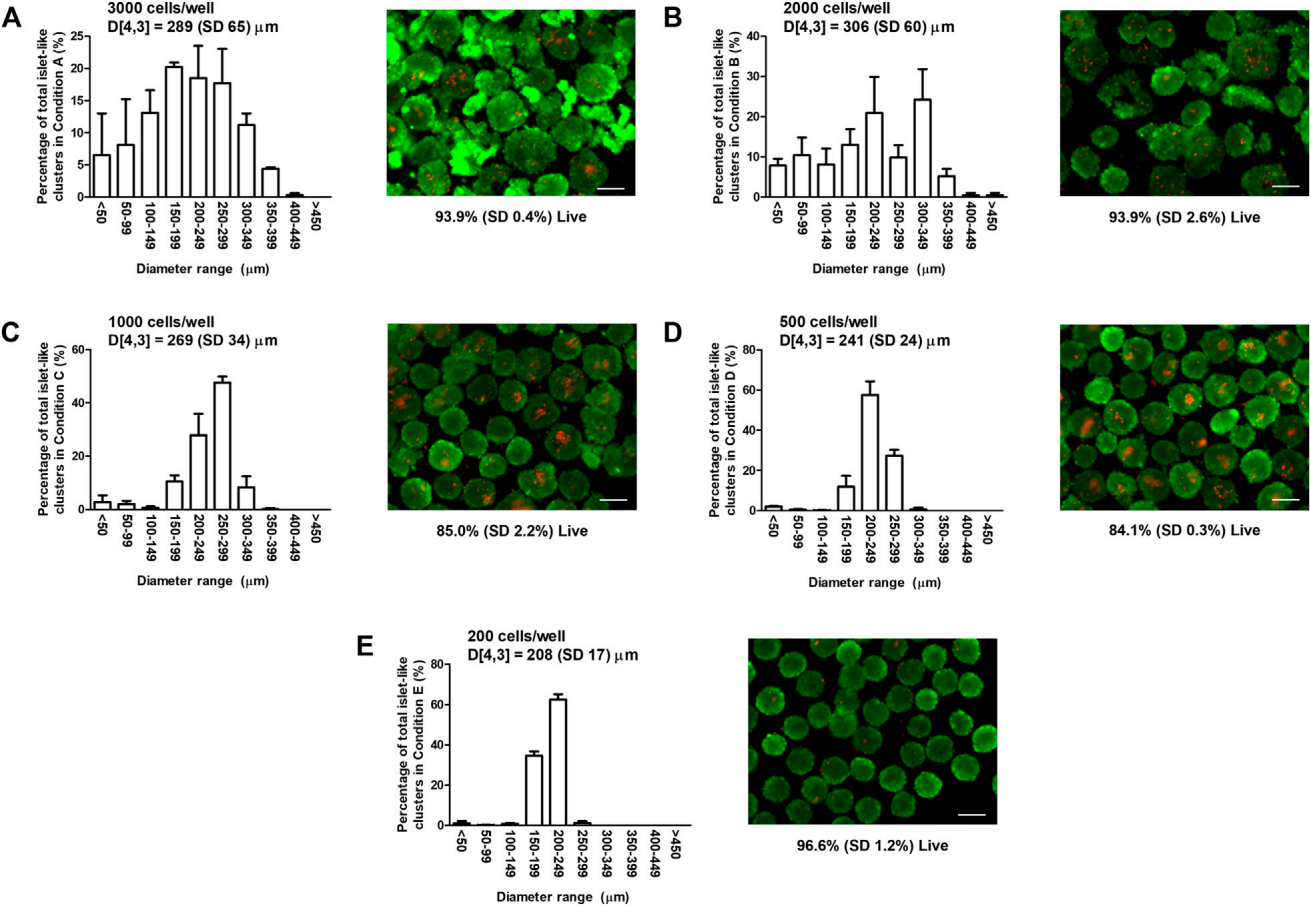
Mouse insulinoma 6 (MIN6) aggregate size distribution and viability. Bar graphs show the size distribution and volume mean moment (De Brouckere mean diameter) D [4,3] with standard deviation SD of MIN6 islet-like clusters (ILCs) generated using AggreWell™400 microwell plates. Fluorescence images show representative live/dead staining results using calcein-AM (green) and ethidium homodimer-1 (red), from which the mean percentage of live cells is determined. **(A)** Seeding density = 3,000 cells/well, D [4,3] = 289 (SD 65) µm, viability = 93.9% (SD 0.4%); **(B)** Seeding density = 2,000 cells/well, D [4,3] = 306 (SD 60) µm, viability = 93.9% (SD 2.6%); **(C)** Seeding density = 1,000 cells/well, D [4,3] = 269 (SD 34) µm, viability = 85.0% (SD 2.2%); **(D)** Seeding density = 500 cells/well, D [4,3] = 241 (SD 24) µm, viability = 84.1% (SD 0.3%); and **(E)** Seeding density = 200 cells/well, D [4,3] = 208 (SD 17) µm, viability = 96.6% (SD 1.2%). N = 3 biological replicates with 30 images (>400 ILCs) analyzed per condition. Error bars represent the standard deviation. Viability results were analyzed using a one-way analysis of variance and Tukey multiple comparisons post-hoc test. Conditions **(C,D)** were significantly different from Conditions **(A,B,E)** (*p* < 0.001). All other combinations were not significantly different (*p* > 0.05). Scale bar = 200 µm.

To determine appropriate ILC seeding conditions for perfusion device studies, MIN6 cells were seeded at varying densities in 24-well AggreWell™400 plates and visualized using live/dead staining and fluorescence microscopy. Size distributions and representative staining images are shown in Figure 1. The bar graphs in Figure 1 indicate that the ILC size distribution narrows as the seeding density decreases. The volume mean moment (De Brouckere mean diameter) D [4,3] calculated for each condition ranges from 208 to 306 µm. Conditions A (3,000 cells/ILC) and B (2,000 cells/ILC), exhibit broad size distributions, with >14% of total ILCs measuring <100 µm in diameter. ILCs in Conditions A and B are also visibly irregular in shape when contrasted to imaging results from Conditions C–E. This is likely due to an overflow of excess cells in the well plates, causing inadequate aggregation and irregular clumping. D [4,3] decreases from Condition B through Condition E, as does the corresponding standard deviation.

Overall cell survival for each condition was quantified using live/dead staining images. Adherent MIN6 controls were also stained and analyzed (images not shown), resulting in a mean viability of 96.8%. For MIN6 ILCs, the mean viability ranges from 84.1 to 96.6%. No significant difference is observed between the viability of the control and the ILCs at Conditions A, B, and E (*p* > 0.05). Significant differences are observed between the control and Conditions C and D (*p* < 0.001). Viability is observed to be higher in Conditions A and B likely because of the broad size distribution and relatively high proportion of small ILCs (<150 µm diameter) experiencing high mass transport. Similarly, viability is high in Condition E and is attributed to the uniformly smaller ILCs (D [4,3] = 208 µm).

### Oxygen Consumption Rate of MIN6 Islet-like Clusters

**FIGURE 2:**
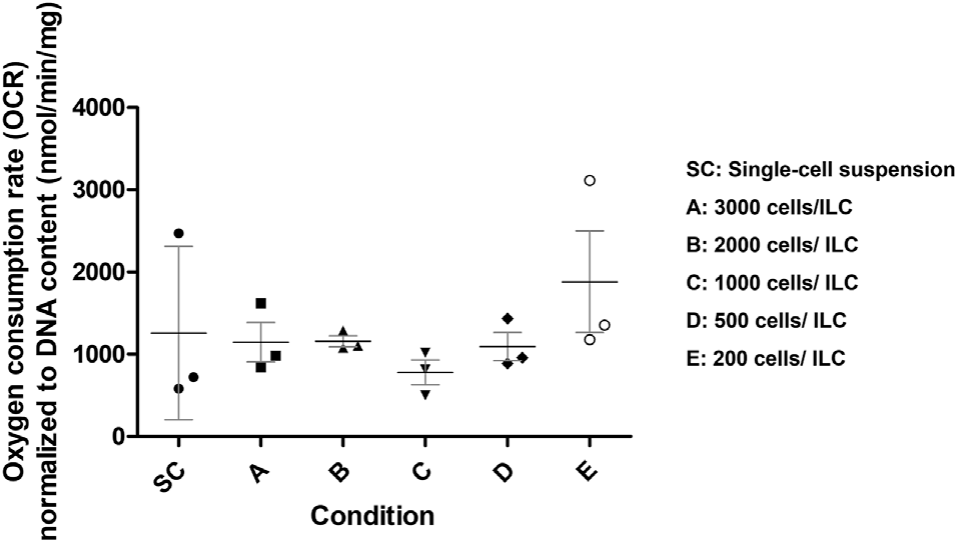
Mouse insulinoma 6 (MIN6) oxygen consumption rate (OCR) normalized to DNA content. OCR was measured for MIN6 islet-like clusters (ILCs) generated using different seeding densities in AggreWell™400 microwell plates. Conditions are SC = single-cell suspension (control); A = 3,000 cells/ILC; B = 2,000 cells/ILC; C = 1,000 cells/ILC; D = 500 cells/ILC; E = 200 cells/ILC. Using a one-way analysis of variance, no significance difference was observed between the means of conditions (*p* = 0.5041). N = 3 biological replicates. Error bars represent the standard deviation.

To further assess appropriate ILC seeding conditions, the oxygen consumption rate (OCR) was measured for Conditions A–E and normalized to DNA content as presented in Figure 2. A breakdown of the numerical results is also available in Supplementary Table S1. Single-cell MIN6 controls were also analyzed. Using a one-way ANOVA, no significant difference was observed between the means of the conditions, although it was noted that the standard deviations were smallest for Conditions B–D. From the literature, human research islets cultured with 10% fetal bovine serum exhibit an OCR/DNA of 202 (SD 87) nmol/min/mg (Papas et al., 2007a). The OCR values measured for MIN6 ILCs are therefore 4–6 times higher than in human research islets.

Given the size distribution, viability, and OCR data collected, Condition C was selected as the most appropriate ILC seeding density for future studies. The density of 1,000 beta cells per aggregate best approximates the beta cell content of native human islets (Pisania et al., 2010) while the relatively narrow size distribution facilitates computational modelling parameters. Moreover, the indication of central necrosis occurring in the ILCs (Figure 1C, right) increases the likelihood of observing a measurable response to hypoxia during oxygen studies. Mean OCR normalized to DNA content for Condition C is 780 (SD 261) nmol/min/mg. At a low concentration of 1,000 ILCs/mL gel, this corresponds to an equivalent of approximately 70,000 human islets per device, with each device having a capacity of 18 mL cell-laden gel.

### Computational Modelling Parameters and Parametric Analysis

**FIGURE 3:**
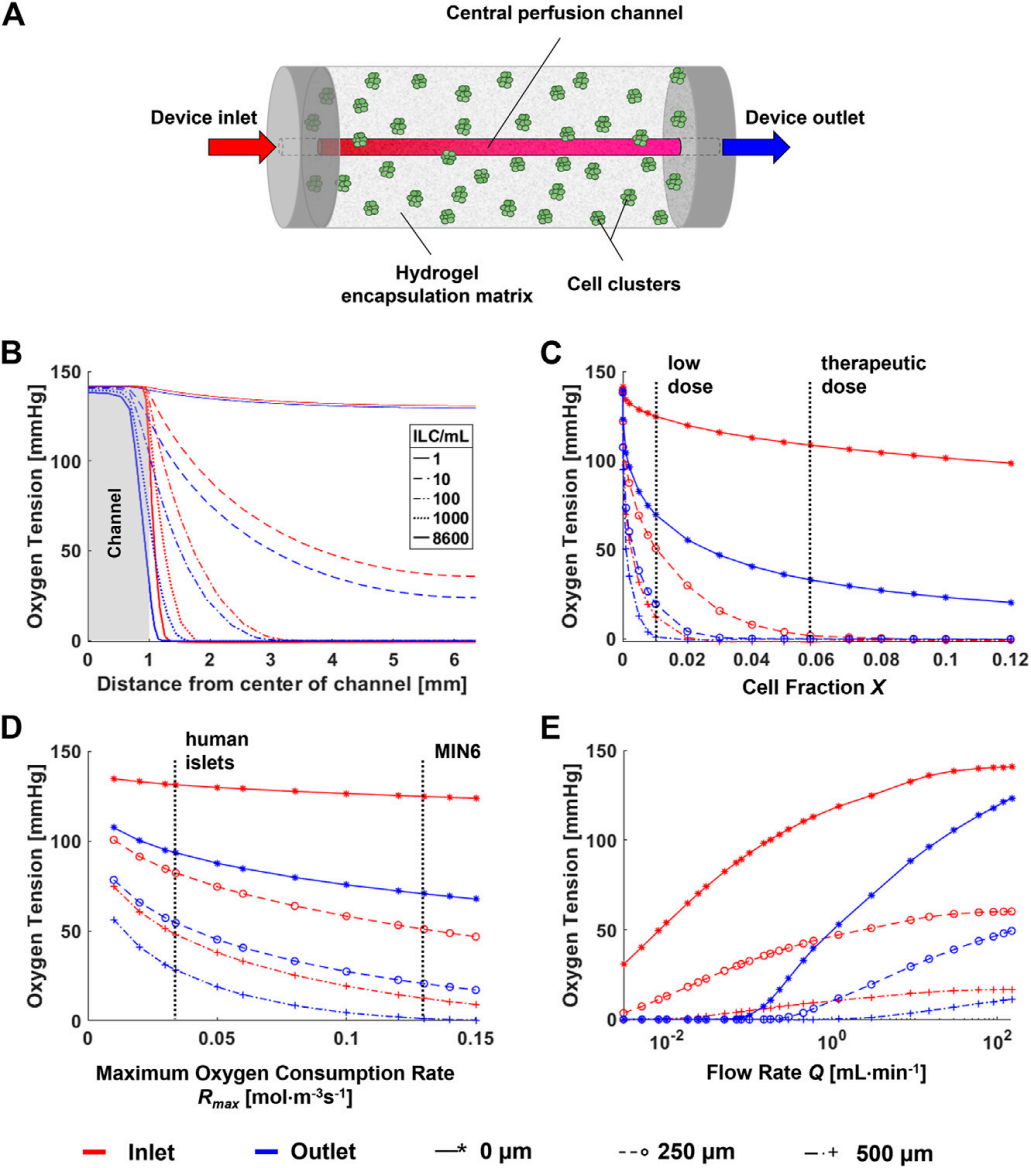
Parametric analysis for a computational model of a single-channel macroencapsulation device. **(A)** Schematic of the major device components. Cell clusters are immobilized in a hydrogel encapsulation matrix featuring a central perfusion channel. **(B)** Radial oxygen profiles at varying cell densities within the device. **(C)** Sensitivity of the model to cell fraction *X*. Vertical lines indicate “low” (1,000 ILCs/mL) and “therapeutic” (8,600 ILCs/mL) doses of MIN6 islet-like clusters (ILCs). The therapeutic dose is calculated by scaling a therapeutic dose of human islets to an equivalent number of MIN6 ILCs based on oxygen consumption rate (OCR). **(D)** Sensitivity of the model to the maximum oxygen consumption rate *R*_max_. Vertical lines indicate the oxygen consumption rate of MIN6 ILCs (obtained experimentally) and human islets (from literature) (Papas et al., 2007a). **(E)** Effect of flow rate on the concentration profile. The colour legend for all panels and data legend for panels **(C–E)** are located at the bottom of the figure.

To predict the oxygen profiles within the perfusion device, a computational model was developed simulating the device geometry, which is a cylinder with length 110 mm, diameter 12.7 mm, and central perfusion channel diameter 2 mm (Figure 3A). Parameters were obtained both from literature and from experimental results as summarized in Table 1.

To assess the effect of the major parameters on the final oxygen concentration profiles, parametric analyses were performed as shown in Figures 3B–E. Points were selected at the device entry and exit at radial distances of 0, 250, and 500 µm from the perfusion channel to observe any notable effects at different positions. Vertical dashed lines on the plots indicate ranges or points of interest with respect to each parameter. Both cell fraction *X* (Figure 3C) and maximum oxygen consumption rate *R*_max_ (Figure 3D) have a significant impact on device oxygen tensions. This occurs within the range of interest for islet transplantation applications. For example, changing from a low cell dose to a therapeutic dose may shift the oxygen tension by several orders of magnitude. Similarly, a change in cell type from human islets to MIN6 causes an overall decrease in oxygen tension due to an increase in oxygen consumption.

Large changes in diffusivity *D* may cause large changes in oxygen tension (data not shown). However, in practice, hydrogels such as alginate tend to have similar diffusivity values at the concentrations used for cell encapsulation (Mehmetoglu et al., 1996; Buchwald, 2011). Therefore, experimental changes in alginate concentration are not expected to significantly affect device oxygen profiles. For changes in *X* and *R*_max_, notable differences are observed between the trends at the device entry and exit, especially with increasing total oxygen consumption in the device. A major difference between entry and exit is observed for changes in flow rate *Q* at low values (Figure 3E). At the experimental flow rate of 3 mL/min, we would anticipate a slight decrease in oxygen tensions along the length of the device. Larger decreases are anticipated at flow rates below 1 ml/min. A similar plot representing the parametric analysis of the dimensionless Reynolds number Re is found in Supplementary Figure S1.

Given these results, immediate applications of this model would focus on the effects of cell type (i.e., OCR) and cell concentration, which significantly influence the oxygen distribution. Future work should further investigate longitudinal profiles within the device.

### Perfusion Culture of Macroencapsulated ILCs Under Varying Oxygen Tensions

**FIGURE 4:**
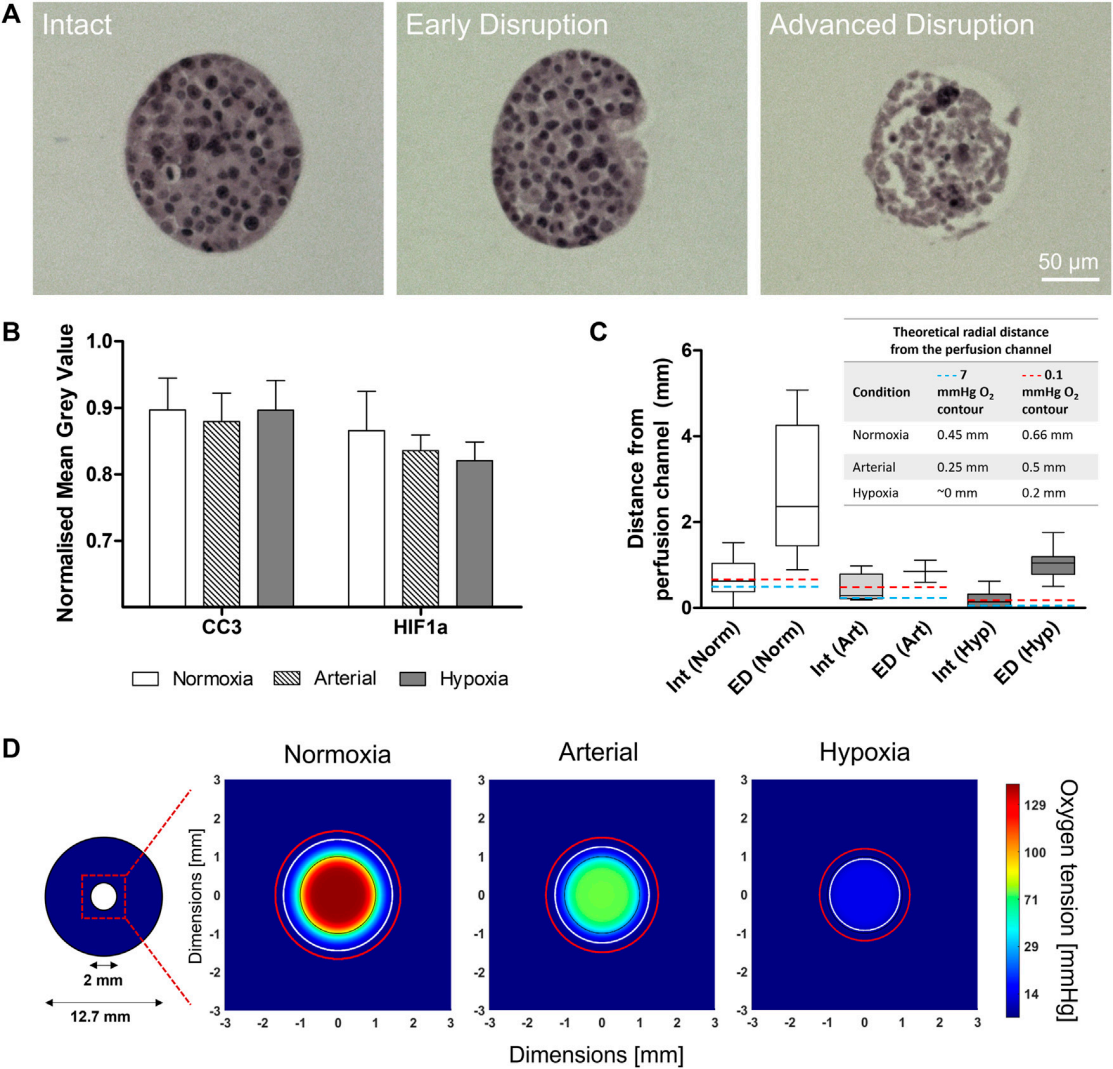
Macroencapsulated MIN6 behaviour after 48-h normoxic (140 mmHg O_2_), arterial (76 mmHg O_2_), and hypoxic (15 mmHg O_2_) perfusion culture. **(A)** Hematoxylin and eosin (H&E) stained sections show the range of islet-like cluster (ILC) morphologies observed: Intact (smooth, rounded), Early Disruption (partial breakage), and Advanced Disruption (pervasive breakage). Scale bars = 50 µm. **(B)** Cleaved caspase-3 (CC3) and hypoxia inducible factor-1α (HIF-1α) staining was performed to assess apoptosis and response to hypoxia, respectively. The average mean grey value (inversely proportional to positive staining intensity) was quantified for all intact ILCs and normalized to the image background. Error bars represent the standard deviation. **(C)** MIN6 ILC morphology relative to distance from the perfusion channel. A box-and-whisker plot illustrates the distribution of Intact and Early Disruption ILCs for each oxygen condition. Abbreviations: Int = Intact, ED = Early Disruption, Norm = Normoxia (140 mmHg O_2_), Art = Arterial (76 mmHg O_2_), Hyp = Hypoxia (15 mmHg O_2_). N > 5 ILCs for each category except Early Disruption (Arterial), where n = 2. The whiskers extend from the minimum to the maximum distance measured from the perfusion channel. Total perfusion culture data is collected from N = 3 (arterial), N = 4 (hypoxia), and N = 5 (normoxia) independent biological replicates. The embedded table indicates the position of the theoretical 7 mmHg (blue dashed line) and 0.1 mmHg (red dashed line) O_2_ contours determined from computational simulations. **(D)** Simulations of the 48-h perfusion experiments in **(A**–**C)**, performed using COMSOL Multiphysics software. Images represent cross-sections taken near the middle of the perfusion device at steady state. The 2-mm perfusion channel is outlined in black at the centre of the cross-section. The colour legend indicates oxygen tension in mmHg. The white contour represents an oxygen tension of 7 mmHg and the red contour represents 0.1 mmHg, corresponding to the positions listed in the table in **(C)**.

Based on the results shown in Figure 1 and Figure 2, MIN6 cells were seeded at Condition C (1,000 cells/ILC), encapsulated in alginate at a concentration of 1,000 ILCs/mL gel, and cast in perfusion devices. Devices were cultured under DMEM perfusion for 48 h at normoxic (140 mmHg or 18.6% O_2_), arterial (76 mmHg or 10% O_2_), and hypoxic (15 mmHg or 2% O_2_) environmental conditions. Samples were then fixed and stained for hematoxylin and eosin (H&E; general tissue visualization), anti-cleaved caspase-3 (CC3; apoptosis), and anti-hypoxia inducible factor-1α (HIF-1α; response to hypoxia). Using H&E-stained sections, ILC morphology was categorized as Intact, Early Disruption, or Advanced Disruption, as illustrated in Figure 4A. Intact (Int) ILCs are smoothly rounded and lack any visible breakage. Early Disruption (ED) samples exhibit limited breakage, typically visible at the ILC periphery or indicated by small holes in the ILC interior. Advanced Disruption (AD) is characterized by pervasive ILC breakage including large interior holes, elongated cells, and peripheral cell debris. Intact ILCs were analyzed for CC3 and HIF-1α staining intensity (Figure 4B). While no statistical differences were observed between conditions, the HIF-1α results suggest a possible increasing trend in positive staining with decreasing oxygen tension. Increased expression of the HIF-1α transcription factor would indicate a response to hypoxia comprising an upregulation in processes such as angiogenesis to promote oxygen delivery.

Intact (Int) and Early Disruption (ED) ILCs were analyzed with respect to distance from the perfusion channel (Figure 4C). Advanced Disruption (AD) samples were excluded as the extreme breakage prevented reliable measurements. Under normoxic conditions, Int and ED ILCs are distributed over a notably larger distance (>5 mm) than observed under arterial and hypoxic conditions (both <2 mm). Under hypoxia, Intact ILCs were rarely observed and severely limited to <0.6 mm from the channel. This aligns with the computational simulation in Figure 4D, which illustrates the radial oxygen distribution under each oxygen condition. In each simulation, a white contour indicates an oxygen tension of 7 mmHg (∼1% O_2_). As impairments in beta cell function have been observed below 7 mmHg (Papas et al., 1996), this value has been used as a lower threshold in previous models (Fernandez et al., 2014). Contrasting normoxia, arterial, and hypoxia simulations, the approximate position of this contour shifts from 0.45 mm to 0.25 mm to ∼0 mm from the perfusion channel, respectively. Similarly, a red contour representing 0.1 mmHg O_2_ shifts from 0.66 mm to 0.5 mm to 0.2 mm from the perfusion channel, respectively. Current literature suggests a threshold of 0.1 mmHg O_2_ for necrosis, implying islets beyond this contour would be dead and no longer consuming oxygen (Johnson et al., 2009; Buchwald, 2011). For all experimental conditions in Figure 4C, beyond the range of ED ILCs, the remaining distance to the device periphery was populated solely with AD ILCs (not quantified due to pervasive breakage).

## Discussion

This work demonstrates the impact of perfusion and oxygen dynamics on the integrity and viability of alginate-immobilized islet-like clusters (ILCs). Further, supplementing these experimental applications with computational simulations is proposed to streamline the device optimization process. While numerous islet encapsulation designs are being explored to treat type 1 diabetes, there remains a gap in successful translation from benchtop to bedside—major obstacles being graft hypoxia and other mass transport limitations. Rigorous *in vitro* and *in silico* investigation into oxygen effects can provide valuable insight into engineering design requirements at early stages of development, reducing overreliance on costly and inefficient animal studies.

While beta cell lines such as MIN6 are widely used in diabetes research, systematic characterization of these cells is often lacking. This creates barriers to accurately predicting the performance of human islets and attempting to scale up devices for clinical use. For the perfusion studies described in this work, MIN6 ILCs were generated via microwell plates and characterized with respect to viability, size distribution, and oxygen consumption as shown in Figure 1 and Figure 2. High seeding densities (up to 3,000 cells/ILC) produced broad size distributions, creating challenges to validating early iterations of the computational model. While low seeding densities (down to 200 cells/ILC) exhibited high cell survival, a density of 1,000 cells/ILC was deemed to reflect beta cell content in native islets more closely while also generating ILCs large enough to observe central necrosis and other oxygen effects (Pisania et al., 2010). This seeding density of 1,000 cells/ILC was selected for further studies and corresponds to a mean diameter of 269 (SD 34) µm, 85% (SD 2%) viability, and an OCR of 780 (SD 261) nmol/min/mg. For comparison, primary islet size ranges from <50 µm to >350 µm in diameter, though most of the volume is made up of islets >150 µm in diameter (Lehmann et al., 2007; Suszynski et al., 2014). The human islet OCR reported in literature (Papas et al., 2007a) is 3.86 times lower than the measured MIN6 OCR. This observation is not surprising given that primary beta cells are non-dividing while alginate-immobilized MIN6 cells exhibit doubling times of 3.6 days (Hoesli et al., 2011). This is coupled with the known correlation between cell growth and OCR (Shuler and Kargi, 2002).

Similar characterization methods could be applied to other beta cell lines including pluripotent stem cell-derived islets to better inform parameter selection. Knowing the OCR, cell density may be scaled appropriately to represent a desired equivalent of human islets. While ILC preparation and harvesting via microwell plates are relatively simple and maintain high cell viability, this method limits production to <30,000 ILCs per plate. Therefore, repeated studies involving high cell concentrations (e.g., >100,000 ILCs per device) may incur higher costs and long harvesting times due to the large number of plates required. Alternative ILC generation methods, such as growing cell clusters in a 3D gel scaffold, may be worth further investigation for scale-up purposes (Hoesli et al., 2009).

The single-channel perfusion strategy employed in this work facilitates investigation of a radial oxygen distribution through the cylindrical macrocapsule. The histology results in Figure 4C show changes in ILC morphology with respect to perfusion channel proximity. Under normoxic culture conditions, Intact ILCs are observed over a distance of nearly 2 mm from the perfusion channel. The distribution of Early Disruption ILCs extends over a 4-mm distance, including the peripheral regions of the gel. In contrast, both Intact and Early Disruption ILCs under arterial and hypoxic conditions are limited to within approximately 2 mm of the perfusion channel, beyond which only Advanced Disruption ILCs are observed. Given that such differences were observed using a relatively low ILC concentration equivalent to approximately 70,000 IEQ/device, this stresses the importance of mimicking relevant *in vivo* conditions during early development of encapsulation devices. Notably, several subcutaneous islet devices relying on passive oxygen diffusion, such as ViaCyte’s PEC-Encap™, have advanced to human clinical trials (ClinicalTrials.gov Identifier NCT04678557). However, such strategies are susceptible to forming large hypoxic zones—particularly when sized to accommodate a full therapeutic dose of islets. Even devices designed to permit endogenous vascularization may not suffice, as hypoxic zones form over a much shorter timescale (hours) than needed for *in vivo* neovascularization to occur (weeks) (Jones et al., 2007). This would indicate that artificial vascularization, device prevascularization (e.g., Defymed’s MailPan^®^ device), or co-encapsulation of oxygen carriers/generators (e.g., Wang et al.‘s inverse-breathing encapsulation device) could prove more promising to address mass transfer limitations at the human scale (Magisson et al., 2020; Wang et al., 2021).

Figure 4B shows that staining for apoptosis (anti-CC3) and response to hypoxia (anti-HIF-1α) among Intact ILCs did not vary significantly across the different oxygen culture conditions. It is reasoned that apoptotic cells are more highly represented among Early Disruption and Advanced Disruption ILCs. However, positive staining is difficult to quantify in these categories due to pervasive ILC breakage. The HIF-1α staining results suggest an increasing trend in positive staining (i.e., increased response to hypoxia) as the environmental oxygen tension decreases, as would be expected. Future studies should investigate this more effectively by increasing the ILC density in the device, thereby increasing the overall oxygen consumption and generating stronger oxygen gradients. Higher ILC densities will also be required to mimic a full therapeutic dose of primary islets (∼600,000 IEQ per patient delivered into the portal system) (Buder et al., 2013; Zorzi et al., 2015). As previously discussed, alternative ILC generation methods could facilitate this scale-up.

Computational modelling techniques are underused in the field of islet encapsulation but may contribute greatly to the accurate prediction of *in vivo* device performance. As a first step, we created a simple model replicating the single-channel perfusion device geometry. Using experimental and literature values to establish the model parameters, oxygen concentration profiles were successfully simulated with fluid convection at different oxygen tensions (Figure 4D). The experimental results in Figures 4A–C were found to generally agree with the trends observed in the simulations, further emphasizing the need for active oxygenation under *in vivo* conditions. Interestingly, Intact ILCs were observed at distances beyond the predicted contours for functional impairment and necrosis, suggesting that MIN6 cells may be more resilient than anticipated. In future work, this model will be further developed to increase complexity, such as simulating discrete ILCs or creating heterogeneity across the device. Importantly, the model may be used to estimate the required density and geometry of microvasculature to adequately nourish the cells. Simulations could thus prove instrumental in device optimization, enabling an iterative approach between experimental and computational work to improve the oxygen distribution.

**FIGURE 5:**
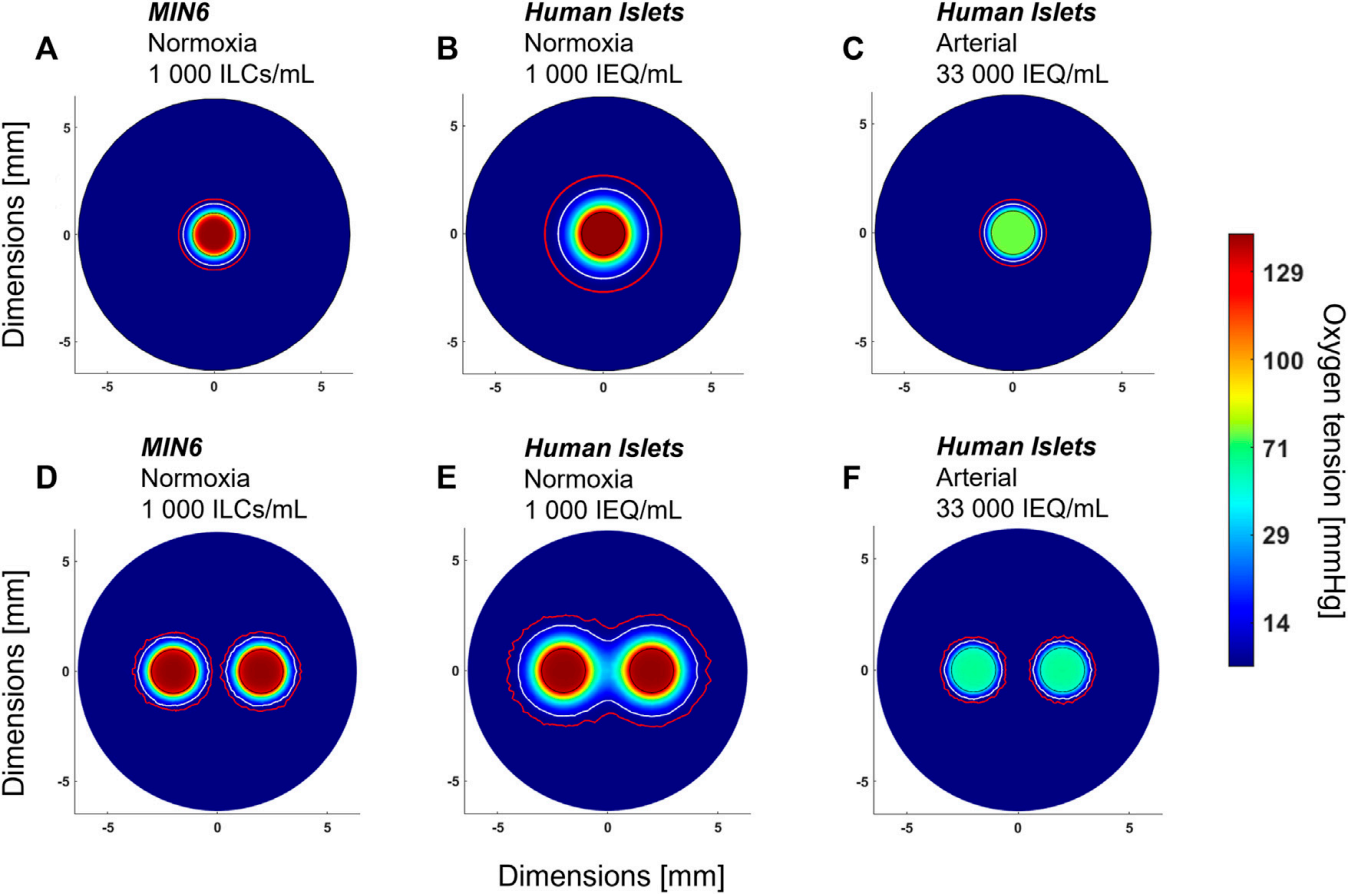
Anticipated effect of device design parameters on oxygen distribution. All images shown are radial cross-sections taken at the middle of the device at steady state. **(A)** Mouse insulinoma 6 (MIN6) islet-like clusters (ILCs) encapsulated in 2% alginate at a concentration of 1,000 ILCs/mL gel and perfused with cell culture medium under normoxic conditions (140 mmHg O_2_). **(B)** Same parameters as **(A)** but substituting MIN6 ILCs with human islets exhibiting an oxygen consumption rate of 202 ± 87 nmol/min/mg (Papas et al., 2007a). **(C)** Same parameters as **(B)** but cell density increased to a therapeutic dose of 600,000 IEQ per device and perfused at an arterial oxygen tension (76 mmHg O_2_) (Buder et al., 2013). **(D)** Same parameters as **(A)** with an arrangement of two perfusion channels, each 2 mm in diameter and spaced 2 mm apart. **(E)** Same parameters as **(B)** with an arrangement of two perfusion channels. **(F)** Same parameters as **(C)** with an arrangement of two perfusion channels. In each panel, a white contour represents an oxygen tension of 7 mmHg and a red contour represents 0.1 mmHg O_2_. The colour legend indicates oxygen tension in mmHg O_2_. Islet equivalent, IEQ = islet of diameter 150 µm.

Using the current model, parametric analyses in Figure 3 indicate that cell density and OCR strongly influence oxygen profiles and can be used to predict the outcome of changing these parameters. As mentioned above, an additional parameter of interest is the number of perfusion channels present in the macroencapsulation device. While the current device is designed to accommodate a single, central channel, the results in Figure 4 highlight that even at normoxia, this is insufficient to avoid oxygen limitations near the device periphery. Therefore, important future work involves accommodating and optimizing a multichannel network to increase fluid convection within the cell-laden gel. Currently, different parameter settings are modelled as shown in Figure 5 to illustrate the challenges and provide an example of possible future device optimization.

As a starting point, Figure 5A shows the radial oxygen profile for one of the experimental conditions used in Figure 4—1,000 MIN6 ILCs/mL alginate gel perfused at 3 mL/min under normoxic conditions (140 mmHg O_2_). Figures 5B,C illustrates the predicted oxygen distributions when substituting MIN6 cells with human islets, which have an oxygen consumption rate approximately four times lower than that of MIN6 ILCs. At a low seeding density (1,000 islet equivalents (IEQ)/mL gel), there is expected to be adequate oxygenation over a distance of ∼1 mm from the perfusion channel (Figure 5B). However, assuming *in vivo* transplantation conditions, i.e., a human therapeutic dose of 600,000 IEQ per device under arterial oxygen tensions, device oxygenation becomes far more restricted (Figure 5C). Figures 5D–F illustrates how these oxygen distributions could change with the addition of a second perfusion channel. Notably, Figure 5E highlights how a multichannel approach could reduce hypoxic zones. These predictions provide insight into encapsulation device requirements to effectively use native blood flow for oxygenation. It is important to note that increasing the number of channels reduces the device volume occupied by cell-laden gel, thereby necessitating higher cell densities. By considering different channel numbers and geometric arrangements *in silico*, an optimal trade-off between cell density and vascular complexity may be approached.

This work describes a versatile platform to assess and validate engineering assumptions and physical properties relevant to a variety of islet encapsulation approaches. While a simple, cylindrical model was selected as a starting point, the proposed combination of experimental studies and computational simulations may be adapted to different device geometries that incorporate artificial vasculature. More generally, computational modelling is a powerful tool that can streamline the development of active oxygenation strategies. Outside of islet device applications, the results of this work are relevant to a broader range of cell therapy devices and immobilized cell culture systems where oxygen limitations remain a major roadblock. Advancing a robust *in vitro* platform integrating fluid convection could permit more rigorous investigation into immobilized cell interactions with implications in both fundamental and translational research. Developing an iterative approach between computational and experimental methods offers high potential for optimizing diverse bioengineering systems and increasing the strength of early development biomedical research.

## Data Availability

The raw data supporting the conclusion of this article will be made available by the authors, without undue reservation.
