# Supplementary material for: Engineering Vascularized Islet Macroencapsulation Devices: An in vitro Platform to Study Oxygen Transport in Perfused Immobilized Pancreatic Beta Cell Cultures
Source: Front Bioeng Biotechnol. 2022 Apr 19;10:884071. doi: 10.3389/fbioe.2022.884071 (PMC9061948; doi:10.3389/fbioe.2022.884071)
Supplement: Supplementary file 1 [file DataSheet1.pdf]

Supplementary Material

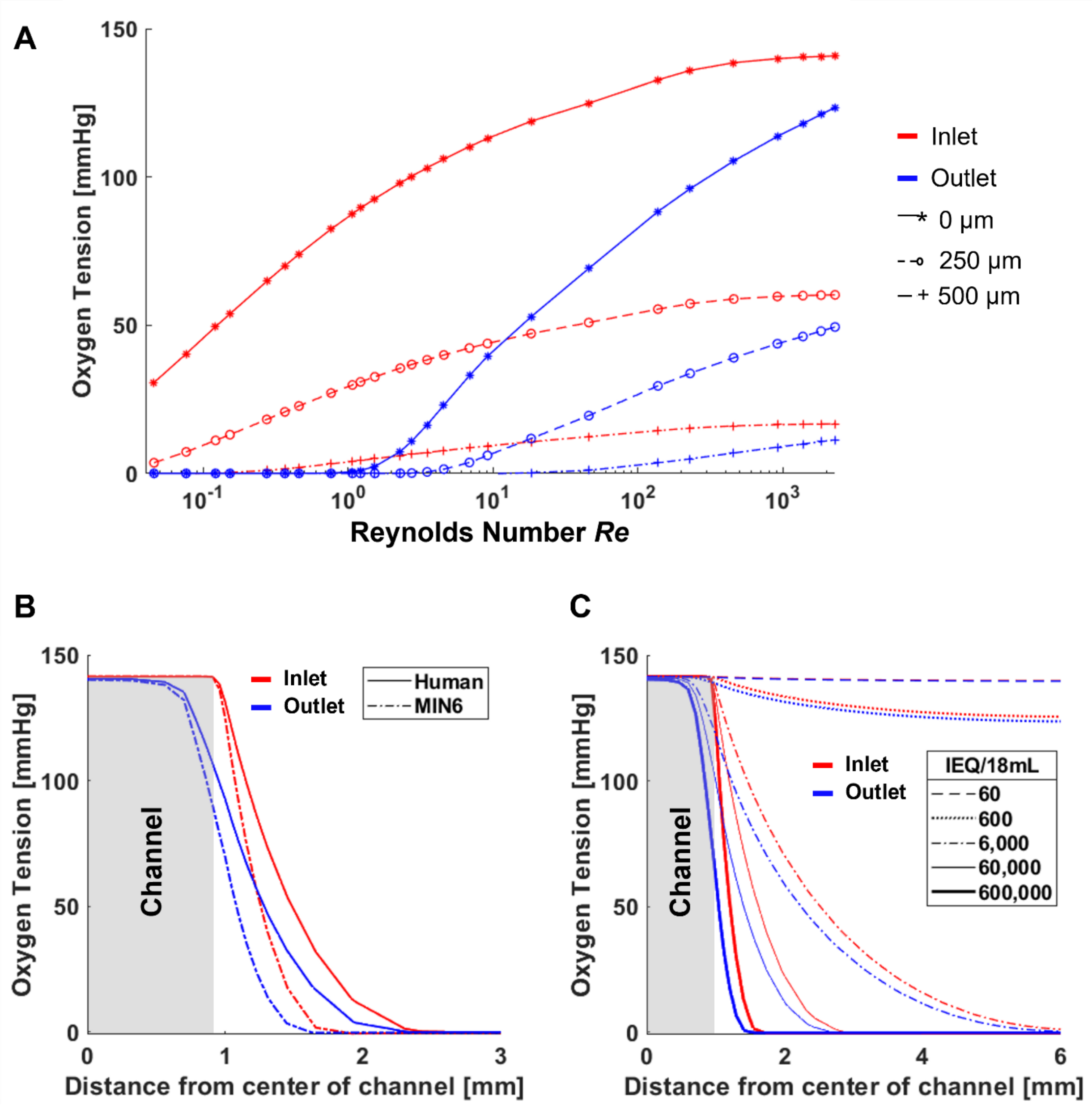

Supplementary Figure 1: Additional parametric analysis for a computational model of a single-channel macroencapsulation device. (A) COMSOL Multiphysics software was used to determine the effect of the Reynolds number *Re* on the device oxygen distribution. Six points were selected at the device entry and exit at radial distances of 0, 250, and 500 µm away from the perfusion channel to observe both radial and lengthwise changes across the device. **(B)** Comparison of simulated radial oxygen profiles for MIN6 and human islets. **(C)** Comparison of simulated radial oxygen profiles for different concentrations of human islets. Islet equivalent, IEQ = islet of diameter 150 µm.

Supplementary Table 1: Oxygen consumption rate of mouse insulinoma 6 (MIN6) islet-like clusters (ILCs).

Oxygen consumption rate (OCR) normalized to DNA content [nmol/min/mg]					
**Sample**	**Replicate 1**	**Replicate 2**	**Replicate 3**	**Mean**[†]	**Standard Deviation**[‡]
**Single-cell suspension**	581	723	2472	1259	1053
**Condition A**<br><br>**3000 cells/ILC**	1622	840	984	1149	416
**Condition B**<br><br>**2000 cells/ILC**	1288	1078	1106	1158	114
**Condition C**<br><br>**1000 cells/ILC**	503	814	1021	780	261
**Condition D**<br><br>**500 cells/ILC**	960	1436	889	1095	297
**Condition E**<br><br>**200 cells/ILC**	1355	1178	3116	1883	1072

[†]No significant difference found between the means using a one-way analysis of variance (ANOVA), $\alpha = 0.05$.
[‡]No outliers identified using the two standard deviation rule.
